# Altered Gamma-Band Activity as a Potential Biomarker for the Recurrence of Major Depressive Disorder

**DOI:** 10.3389/fpsyt.2018.00691

**Published:** 2018-12-12

**Authors:** Tetsuya Yamamoto, Nagisa Sugaya, Greg J. Siegle, Hiroaki Kumano, Hironori Shimada, Sergio Machado, Eric Murillo-Rodriguez, Nuno B. Rocha, Antonio E. Nardi, Masahiro Takamura, Yasumasa Okamoto, Shigeto Yamawaki

**Affiliations:** ^1^Graduate School of Technology, Industrial and Social Sciences, Tokushima University, Tokushima, Japan; ^2^Unit of Public Health and Preventive Medicine, School of Medicine, Yokohama City University, Yokohama, Japan; ^3^Department of Psychiatry, University of Pittsburgh School of Medicine, Pittsburgh, PA, United States; ^4^Faculty of Human Sciences, Waseda University, Tokorozawa, Japan; ^5^Physical Activity Neuroscience, Physical Activity Postgraduate Program, Salgado de Oliveira University, Niterói, Brazil; ^6^Laboratorio de Neurociencias Moleculares e Integrativas, Escuela de Medicina, División Ciencias de la Salud, Universidad Anáhuac Mayab, Mérida, Mexico; ^7^School of Health, Polytechnic Institute of Porto, Porto, Portugal; ^8^Panic and Respiration Laboratory, Institute of Psychiatry, Federal University of Rio de Janeiro, Rio de Janeiro, Brazil; ^9^Department of Psychiatry and Neurosciences, Institute of Biomedical and Health Sciences, Hiroshima University, Hiroshima, Japan

**Keywords:** cognitive reactivity, EEG, depression, gamma, mood induction, recovery, biomarker, memory

## Abstract

**Background:** The neurophysiological mechanisms of cognitive reactivity, the primary vulnerability factor of major depressive disorder (MDD) recurrence, remain unclear in individuals with recovered MDD (rMDD). Because gamma-band responses (GBRs) can be used to measure cognitive processing, they may also be useful for elucidating the mechanisms underlying cognitive reactivity. Identifying these mechanisms may permit the development of an index for predicting and preempting MDD recurrence. Here, to identify the neurophysiological mechanisms of cognitive reactivity, we examined the characteristics of the GBRs evoked/induced by emotional words in participants with and without rMDD after inducing a negative mood.

**Methods:** Thirty-three healthy control participants and 18 participants with rMDD completed a lexical emotion identification task during electroencephalography along with assessments of cognitive reactivity after negative mood induction.

**Results:** No between-group differences were identified for the task reaction times; however, the rMDD group had significantly higher cognitive reactivity scores than did the control group. Furthermore, the power of late GBRs to positive words was significantly greater in the rMDD group, with the greater power of late GBRs being related to higher cognitive reactivity.

**Limitations:** Considering the population studied, our findings cannot be completely generalized to populations other than adolescents, people with rMDD, and those without a history of co-morbid disorders and early life stress.

**Conclusions:** Our findings indicate that the dysfunction of neural circuits related to higher-order processes like memory and attention might underlie cognitive reactivity. Altered late GBRs to positive information may be persistent biomarkers of the depression recurrence risk.

## Introduction

Major depressive disorder (MDD), which is characterized by a persistently depressed mood and/or anhedonia, shows a high rate of recurrence after recovery. The recurrence rate in a specialized mental healthcare setting is as high as 85% within 15 years (1), while that in a community-based setting it was found to be ~40% during a 30–39-year follow up (2). To prevent MDD recurrence, it is essential that the mechanisms underlying the associated vulnerability factors be identified. Prior research has shown that the primary vulnerability factor for the recurrence of MDD is cognitive reactivity (3, 4), which is a negative information-processing bias that is triggered when a depressive mood is experienced (5).

Numerous previous studies have reported that people with a history of depression are more likely to demonstrate cognitive biases, such as dysfunctional attitudes (6, 7), biased attention (8, 9), biased interpretation (10, 11), and biased memory (12, 13) after the induction of negative mood. While such cognitive reactivity has mainly been assessed using behavioral and subjective measures (14, 15), several findings have highlighted the low sensitivity of these measurements (16, 17). As such, the underlying biological mechanisms of cognitive reactivity are yet to be identified. Elucidating these mechanisms in individuals with recovered MDD (rMDD) is particularly critical, because cognitive reactivity is “latent” (18), meaning that it is only apparent after psychological challenges and not under ordinary circumstances. Thus, developing a sensitive index for identifying cognitive reactivity in individuals with rMDD, as well as establishing adequate experimental paradigms to study cognitive reactivity, such as those that induce negative mood and use emotional stimuli, is essential for not only assessing the mechanisms underlying cognitive reactivity but also for identifying a potential marker that could alert clinicians to the possibility of recurrence, allowing them to employ preventative or therapeutic measures.

Toward this end, several authors proposed using a neurophysiological approach, such as electroencephalography (EEG), to evaluate the vulnerability factors for depression (18, 19). Indeed, EEG has been shown to be a sufficiently sensitive method for observing mechanisms that could potentially moderate the risk factors for depression in individuals at a high risk (20–22). Prior research regarding gamma-band activity has shown that gamma-band responses (GBRs) are associated with the integration of incoming information and related processes, including feature binding (23), attention (24, 25), object perception (26), and memory (27, 28). These findings suggest that GBRs reflect more than simple perceptual processing, and thus may serve as a marker of complex cognitive mechanisms (29) and major depression (30). Indeed, GBRs comprise both an “early” evoked response (phase-locked) that reflects perceptual processing and a “late” induced response (non-phase-locked) that reflects higher-order cognitive processing, including memory and attentional processes (27). Hence, these two response types may provide information regarding the presence of abnormalities in perception and cognition in individuals who are vulnerable to depression.

Studies examining patients with schizophrenia and MDD using EEG have found that these patients exhibit different GBR characteristics compared to healthy individuals in response to a standard passive auditory oddball paradigm (31) and to emotional words (32). In these studies, compared to those in healthy controls, the amplitudes of early evoked GBRs were significantly smaller in patients with schizophrenia, but not in patients with MDD (31), while the mean power of sustained induced GBRs (3–4 s following the offset of the stimulus) was significantly larger in patients with MDD and significantly smaller in patients with schizophrenia (32). Together, these studies indicate that GBRs can be used as a novel sensitive neurophysiological index of cognitive reactivity. However, to our knowledge, no study has investigated the GBR characteristics in individuals with rMDD using mood induction. Evaluating GBR characteristics using this approach may contribute to our understanding of the neurophysiological mechanisms of cognitive reactivity and allow us to identify a biomarker of this vulnerability factor in individuals with rMDD.

Therefore, the aim of the present study was to examine the characteristics of GBRs to emotional words after inducing a negative mood in individuals who had recovered from recurrent or single-episode depression and compare them with the GBRs of individuals who had never experienced MDD.

## Materials and Methods

### Participants

Fifty-four participants were recruited to this study through poster advertisements. All participants were carefully screened by experienced clinical psychologists to determine if they had current and/or a history of psychiatric disorders according to the Diagnostic and Statistical Manual for Mental Disorders, Fourth Edition, Text Revision (DSM-IV-TR) (33). Exclusion criteria were left-handedness or ambidextrousness, as determined by the Edinburgh Inventory (34), and a previous history of head injury, psychiatric disorders other than MDD, or severe or acute medical illnesses. In addition, to control for current psychiatric symptoms, participants who met the DSM-IV-TR criteria for an Axis I disorder including MDD at the time of the examination were excluded. Furthermore, because successful negative mood induction was a prerequisite for examining cognitive reactivity, participants who did not show an increase in negative mood or a decrease in positive mood after negative mood induction were excluded. Participants who did not have a sufficient number of uncontaminated EEG trials (< 50%) for analysis after the rejection of artifact-contaminated trials were also excluded. After applying all exclusion criteria, data from a total of 51 participants were included in the final analysis. Of these, 18 participants who met the criteria for previous MDD were assigned to the rMDD group. At the time of the study, participants in the rMDD group had recovered from MDD, meaning that they no longer met the DSM-IV-TR criteria for MDD. The remaining 33 participants were assigned to the control group. The detailed demographic and clinical features of the participants are shown in Table 1. There were no between-group differences in age [*t*_(49)_ = 1.63, *p* = 0.109], sex [χ${}_{\left(1\right)}^{2}$ = 0.04, *p* = 0.850], education [*t*_(49)_ = 0.94, *p* = 0.351], or presence of current depression [*t*_(49)_ = 0.70, *p* = 0.078].

**Table 1:** Demographic and clinical characteristics and behavioral data.

**Measure**	**rMDD (*n* = 18)**		**Control (*n* = 33)**	
	**Mean**	***SD***	**Mean**	***SD***
Age (years)	21.72	5.63	20.03	1.51
Sex (M/F)	5/13		10/23	
Education (years)	14.72	2.02	14.26	1.47
Age of onset of first episode (years)	18.89	5.25	−	
Duration of last episode (months)	7.71	16.68	−	
Single episode/recurrent episode	10/8		−	
No. of persons who experienced an episode in the last year	7		−	
BDI-II	9.72	8.09	6.27	5.55
LEIDS-R total score	47.67	14.62	30.39	14.51
Hopelessness/Suicidality	4.94	3.39	2.36	2.88
Acceptance/Coping	2.33	2.40	2.12	2.22
Aggression	9.44	5.52	6.18	3.26
Control/Perfectionism	5.22	3.04	2.76	2.89
Risk Avoidance	13.00	2.72	9.18	4.82
Rumination	12.72	5.42	7.79	4.11
**PROFILE OF MOOD STATES**				
Happy, pre-mood induction	56.11	16.14	60.03	15.90
Happy, post-mood induction	43.33	23.76	37.88	18.50
Sad, pre-mood induction	34.44	25.49	24.24	19.20
Sad, post-mood induction	40.00	21.69	34.85	22.38
**REACTION TIMES IN THE EVIT (SECONDS)**				
Positive words	0.80	0.15	0.86	0.14
Neutral words	0.85	0.14	0.96	0.20
Negative words	0.81	0.15	0.92	0.17

BDI-II, Beck Depression Inventory II; EVIT, Emotional Valence Identification Task; LEIDS-R, Leiden Index of Depression Sensitivity-Revised

The study was approved by the Ethics Committee of Waseda University. All participants provided written informed consent and received 1,500 Japanese Yen (nearly 14 USD) in remuneration.

### Self-Report Measures

Participants completed two questionnaires prior to the experimental task. These were the (i) Japanese version of the Beck Depression Inventory-II (BDI-II) (35, 36) and (ii) Japanese version of the Leiden Index of Depression Sensitivity-Revised (LEIDS-R) (15, 37).

We employed the BDI-II to assess the depression severity at the time of testing. The BDI-II consists of 21 items that are scored on a Likert scale ranging from 0 (no depressive symptoms) to 3 (strong presence of a symptom). The BDI-II shows good reliability and validity (35, 36).

Here, the LEIDS-R was used to assess cognitive reactivity. This index consists of 34 items that are scored on a Likert scale ranging from 0 (not at all) to 4 (very strongly). Participants indicated whether and how their thinking patterns change when they feel down or are experiencing a low mood. The LEIDS-R has favorable psychometric properties, including adequate internal consistency, test-retest reliability, and concurrent and predictive validity (15). The subscales of the LEIDS-R are Hopelessness/Suicidality, Acceptance/Coping, Aggression, Control/Perfectionism, Risk Avoidance, and Rumination. For this study, we utilized both the total score and the subscale scores of the LEIDS-R.

All participants also assessed their mood before and after mood induction using a visual analog scale (VAS). Participants rated their mood on two unipolar VASs measuring happiness and sadness dimensions. The scales ranged from 0 (not at all) to 100 (extremely).

### Stimuli and Experimental Procedure

#### Mood Induction Paradigm

To induce a negative mood, we used the combination of a 20-anagram task with Japanese nouns (20 solvable) and 20 math tasks in which the participant must solve simple equations (8 solvable/12 unsolvable). This paradigm has been found to successfully induce a depressive mood in healthy people (38). To further increase the mood-inducing effects of this paradigm, participants were informed that most participants get almost all of the answers correct, despite the fact that the math equations were sometimes unsolvable.

#### Emotional Valence Identification Task and Target-Stimulus Materials

We prepared the modified version of the emotional valence identification task (32). First, a fixation cross was displayed for 200 ms at the center of the screen, which was then replaced by an X character string (forward masking stimulus) for a duration of 2,000 ms. Subsequently, the X character string was replaced with a stimulus word (target stimulus), which was displayed for 150 ms. Then, the stimulus word was again replaced with the X character string (backward masking stimulus) for a duration of 8,000 ms, and participants were asked to indicate the emotional valence of the stimulus word by pressing keys corresponding to “positive,” “neutral,” or “negative.” After this initial trial, similar trials were repeated. The stimulus words and masking stimulus were shown in black against a white background. All participants were asked to respond as quickly and accurately as possible. We recorded the reaction time and response type. Notably, the order of stimulus words was randomized, and the keys assigned to the perceived emotional valence determination were counterbalanced.

The 120 stimulus words consisted of 40 depressive, neutral, and positive words, each. Regarding the depressive words, in addition to previously used words (39, 40), we selected words that were closely associated with depression, based on the measurement scales (e.g., BDI-II) and interviews (e.g., the Mini-International Neuropsychiatric Interview) (41) for depressive symptoms that are used in Japan. Regarding neutral and positive words, we selected words according to previous Japanese research findings on emotional words (42). Adjustments were made so that the number of letters and syllables of the words were as similar as possible among the categories.

#### Procedure

Participants were asked to complete the questionnaires (LEIDS-R, VAS, and BDI-II) in a sealed room. After attaching the EEG cap and electrodes, we implemented the mood induction paradigm, and then asked participants to again complete the VAS. Upon completion, participants performed the emotional valence identification task, during which EEG data were recorded. Once the task was finished, we removed the EEG cap and electrodes. Participants were then debriefed. The experiment lasted ~120 min.

### Apparatus and Data Recording

The EEG data were recorded using a Net Amps 200 amplifier, Net Station version 4.2, and a 64-channel HydroCel Geodesic Sensor Net (Electrical Geodesics, Inc., Eugene, OR, USA). All channels were digitized at a sampling rate of 250 Hz and the signal from the electrodes was amplified via the Net Amps 200 amplifier. Recordings were initially referenced to Cz and later converted to an average reference. Impedances were kept below 50 kΩ, which is the recommended impedance threshold for the employed amplifiers (43).

### Data Analysis

#### Behavioral Data

To test the effects of mood induction, we used a three-way repeated measures multivariate analysis of variance (MANOVA), with one between-subjects factor (group: rMDD and control) and two within-subjects factors (mood: happy and sad; time: pre- and post-mood induction). To control for Type I errors across the analyses, we used the Bonferroni correction. The significance level was set at *p* < 0.05 (two-tailed).

Multivariate *t*-tests were used to examine between-group differences in the harmonic mean reaction times of the emotional valence identification task and cognitive reactivity scores (total score and subscale scores of the LEIDS-R). *Post-hoc* comparisons were applied only if Hotelling's *T*^2^ indicated significance. The significance level was set at *p* < 0.05 (two-tailed).

All behavioral analyses were performed using SPSS version 22.0 (SPSS Japan Inc., Tokyo, Japan).

#### Electrophysiological Data

The EEG analysis was conducted using the Net Station software (version 4.2; Electrical Geodesics, Inc.). Data were band-pass filtered at 0.02–100 Hz, notch-filtered at around 50 Hz, and noise removal was performed. The data were split into epochs of 1,200 ms (200 ms before to 1,000 ms after stimulus onset). Blinks, eye movements, and other artifacts (with a voltage exceeding ± 120 μV) were detected based on Net Station's eyeblink and movement detection algorithm (43). The data from bad channels were replaced with interpolated data from the remaining channels using the Bad Channel Replacement tool (43), and artifacts were corrected by applying the Gratton procedure (44). Thereafter, all trials were visually inspected for remaining artifacts and rejected as necessary. In addition to trials with no response, trials with reaction times < 150 ms were rejected, because it is possible that these responses were made without considering the stimulus (45). No between-group differences were identified in the percentage of remaining epochs after epoch rejection (rMDD = 89.35%; control = 85.05%).

To detect and characterize both evoked and induced GBRs, a time-frequency analysis was performed on each epoch using a continuous wavelet transform (20–70 Hz, in 1.0-Hz steps), with Morlet wavelets as basic functions. The power at each frequency from 200 ms to 0 ms before stimulus onset was subtracted for each trial. We selected electrodes in a fronto-central region of interest (F3, F4, Fz, C3, C4, and Cz) to prevent the loss of statistical power. Given the known individual variability in the frequency of oscillatory activity (46), we used each participant's individual max power in the 30–70 Hz range in the analyses of the two following time windows: early GBRs (50–150 ms after stimulus onset) and late GBRs (300–800 ms after stimulus onset) (27).

Further analyses were performed using SPSS version 22 (SPSS Japan Inc.) and PROCESS (47). To test whether the rMDD group showed distinct power characteristics for early and late GBRs for positive and negative words, a three-way repeated measures MANOVA was conducted with one between-subjects factor (group: rMDD and control) and two within-subjects factors (valence: positive, neutral, and negative words; component: early and late GBRs). Greenhouse-Geisser-corrected probabilities are reported in any instance in which the assumption of sphericity was violated. To control for Type I errors across the analyses, we used the Bonferroni correction. The significance level was set at *p* < 0.05 (two-tailed).

To examine the association of GBRs with cognitive reactivity in the rMDD group, we performed moderated multiple regression, with group (rMDD and control) as a predictor, GBRs to a priori-determined positive and negative words as a moderator, the LEIDS-R total score as a dependent variable, and participants' moods (happiness and sadness) after mood induction as covariates. Furthermore, we confirmed that the data were not obstructed by multicollinearity using the variance inflation factor coefficient of independent variables. To demonstrate *post-hoc* probing of moderational effects, the significant interactions were probed by testing the conditional effects of group at three levels of GBRs, including one standard deviation below the mean, at the mean, and one standard deviation above the mean. The significance level was set at *p* < 0.05 (two-tailed).

## Results

### Confirmation of Mood Induction

The group × mood × time repeated MANOVA revealed a significant interaction between mood and time [*F*_(1, 49)_ = 30.89, *p* < 0.001, ηp^2^ = 0.39]. After mood induction, participants demonstrated significantly decreased happiness ratings (*p* < 0.001, Δ = −1.19) and significantly increased sadness ratings (*p* = 0.024, Δ = 0.40) (Table 1).

### Behavioral Data and Cognitive Reactivity

Table 1 shows the harmonic mean reaction times and LEIDS-R scores. The multivariate *t*-test for harmonic mean reaction times did not identify a significant between-group difference [Hotelling's *T*^2^ = 0.14, *F*_(3, 47)_ = 2.19, *p* = 0.10, ηp^2^ = 0.12]. In contrast, a similar analysis performed for LEIDS-R scores revealed a significant between-group difference [Hotelling's *T*^2^ = 0.40, *F*_(6, 44)_ = 2.89, *p* = 0.018, ηp^2^ = 0.28]. Specifically, the rMDD group had a significantly higher total LEIDS-R score (*p* < 0.001, *d* = 1.19), as well as significantly higher scores on most LEIDS-R subscales (Hopelessness/Suicidality: *p* = 0.006, *d* = 0.84; Acceptance/Coping: *p* = 0.753, *d* = 0.09; Aggression: *p* = 0.011, *d* = 0.78; Control/Perfectionism: *p* = 0.006, *d* = 0.84; Risk Avoidance: *p* = 0.003, *d* = 0.91; and Rumination: *p* = 0.001, *d* = 1.07), than did the control group.

### Group Differences in Early and Late Gamma-Band Activity

**Figure 1:**
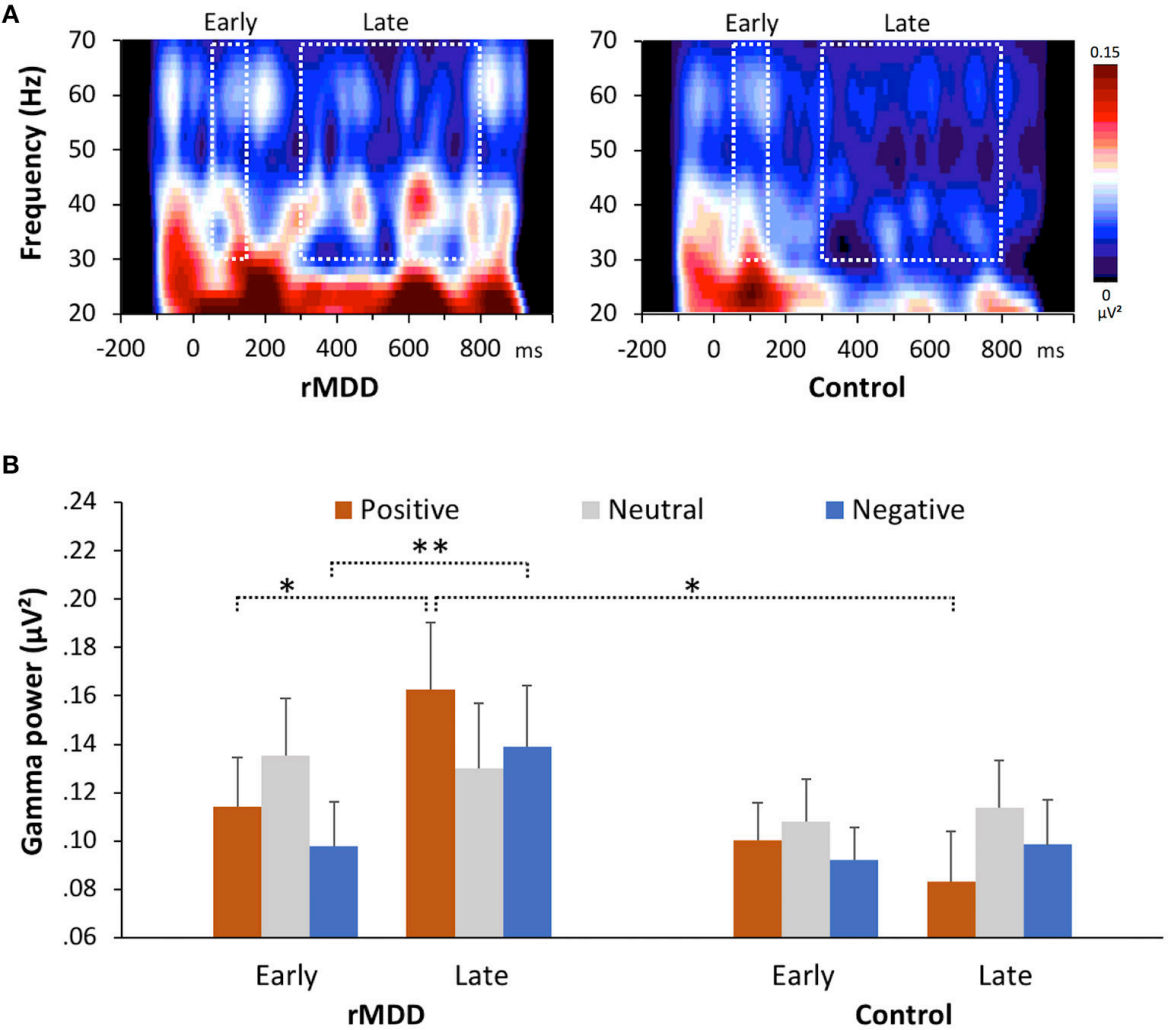
Group differences in gamma-band responses during the emotional valence identification task. **(A)** Wavelet decomposition for both groups showing the response to positive words at all frequencies at electrode F4. **(B)** Early and late gamma powers for all valences in the two groups. Error bars indicate the standard error. *p < 0.05, **p < 0.01, two-tailed.

The group × valence × component repeated measures MANOVA revealed a significant interaction between group and component [*F*_(1, 49)_ = 4.58, *p* = 0.037, ηp^2^ = 0.09] and a significant three-way interaction [Greenhouse-Geisser corrected, *F*_(1.74, 85.44)_ = 6.49, *p* = 0.004, ηp^2^ = 0.12]. The rMDD group showed significantly greater late-GBR power to positive words than did controls (*p* = 0.027, *d* = 0.67), but not to neutral (*p* = 0.623, *d* = 0.14) or negative words (*p* = 0.196, *d* = 0.38), as shown in Figure 1. The rMDD group also showed significantly greater power for late GBRs than for early GBRs to positive (*p* = 0.012, *d* = 0.24) and negative words (*p* = 0.003, *d* = 0.26).

### Association of Gamma-Band Activity With Cognitive Reactivity

**Figure 2:**
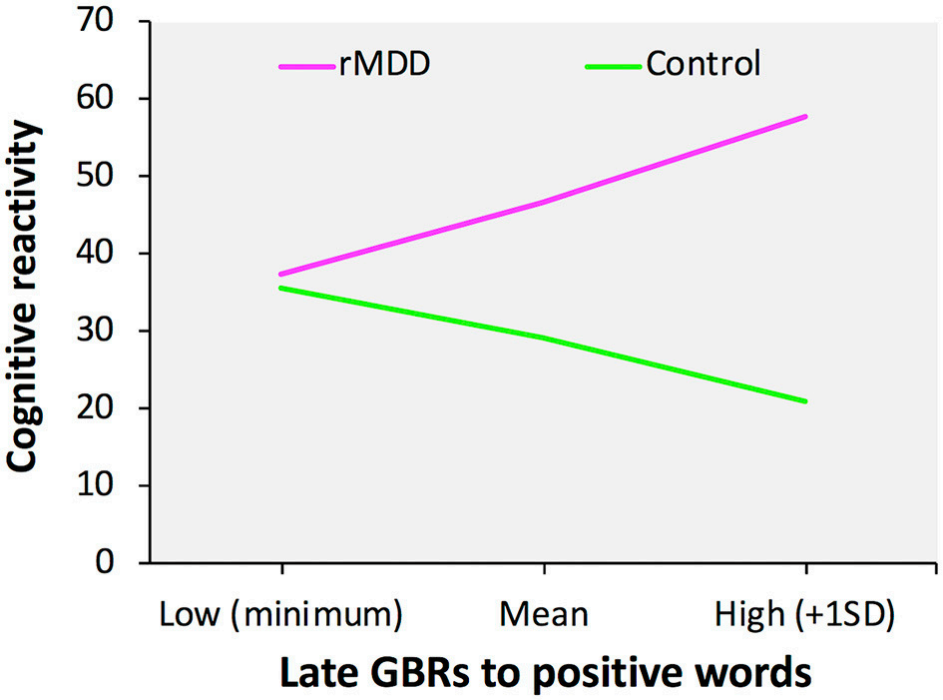
Moderation effects of late gamma-band responses on group and cognitive reactivity. Slope of the relationship between late GBRs to positive words and cognitive reactivity as a function of group.

We confirmed that our data were not obstructed by multicollinearity (variance inflation factor < 6.0). No significant correlations were identified between the LEIDS-R total score and both early and late GBRs: the early GBRs to positive words (*r* = 0.13, *p* = 0.367) or negative words (*r* = 0.04, *p* = 0.789), and the late GBRs to positive words (*r* = 0.14, *p* = 0.322) or negative words (*r* = −0.02, *p* = 0.873). The multiple moderation model was significant when early GBRs were incorporated as a modulator [*F*_(7, 43)_ = 3.99, *p* = 0.002, *R*^2^ = 0.39] as well as when late GBRs incorporated as a modulator [*F*_(7, 43)_ = 4.25, *p* = 0.001, *R*^2^ = 0.41]. We also identified significant interactions between group and late GBRs to positive words [*t*_(43)_ = 2.26, *p* = 0.029, *b* = 155.09] and late GBRs to negative words [*t*_(43)_ = −2.48, *p* = 0.017, *b* = −168.50] (Figure 2), but not early GBRs to positive words [*t*_(43)_ = 2.00, *p* = 0.052, *b* = 202.10] or negative words [*t*_(43)_ = −1.98, *p* = 0.054, *b* = −230.78]. For late GBRs to positive words, group was significantly related to cognitive reactivity when late GBRs to positive words were one standard deviation above the mean [*t*_(43)_ = 3.63, *p* = 0.001, *b* = 36.73] and at the mean [*t*_(43)_ = 3.97, *p* < 0.001, *b* = 17.56], but not when late GBRs to positive words were one standard deviation below the mean [*t*_(43)_ = 0.23, *p* = 0.822, *b* = 1.74]. For late GBRs to negative words, group was significantly related to cognitive reactivity when late GBRs to negative words were one standard deviation below the mean [*t*_(43)_ = 4.17, *p* < 0.001, *b* = 34.79] and at the mean [*t*_(43)_ = 3.97, *p* < 0.001, *b* = 17.56], but not when late GBRs to negative words were one standard deviation above the mean [*t*_(43)_ = −0.04, *p* = 0.967, *b* = −0.35]. As shown in Figure 2, greater late GBRs to positive words and smaller late GBRs to negative words were associated with high predicted cognitive reactivity scores for the rMDD group only.

## Discussion

Identification of the neurophysiological mechanisms underlying cognitive reactivity in individuals with rMDD may provide essential clues regarding the prevention of MDD recurrence. To elucidate these mechanisms, we examined the characteristics of GBRs to emotional words in participants with rMDD and healthy controls after negative mood induction. Our results showed that the mood induction elicited decreased happiness and increased sadness, indicating that this paradigm successfully yielded negative mood changes. After mood induction, although no between-group differences in the reaction times of emotional valence identification were found, the power of late GBRs to positive words was greater in the rMDD group than in the control group. In addition, the rMDD group showed greater power for late GBRs to positive and negative words than for early GBRs, while the control group did not. Furthermore, the rMDD group had significantly higher cognitive reactivity, as measured with the LEIDS-R, than did the control group, and we identified moderation effects of the late GBRs on cognitive reactivity in the rMDD group. Collectively, these findings suggest that individuals with rMDD demonstrate abnormal information processing that influences cognitive reactivity.

To the best of our knowledge, the current study is the first to report altered late induced GBRs during the judgment of positive words in people with rMDD. Because late GBRs are related to the modulation of higher-order cognitive processes, including memory and attention, these GBRs may reflect neuronal synchrony involving large-scale coherence across highly distributed brain regions (48). From this perspective, the between-group GBR differences we identified may be interpreted as neural circuit dysfunction related to the top–down processing of emotional information in rMDD.

The present study also identified significant moderation effects of GBRs, suggesting that participants with rMDD who have a greater power of late GBRs to positive words show high predicted cognitive reactivity scores. These findings indicate that altered processing of incoming positive information in individuals with rMDD who are experiencing a negative mood might exacerbate cognitive reactivity. Our findings support those of previous studies showing that formerly depressed participants exhibit several cognitive biases, including impaired retrieval of contextual details for positive compared to negative events (49, 50), difficulty in using positive memories for emotion regulation (51), and less attentional bias for positive stimuli (52), all of which have been proposed as risk factors for the recurrence of MDD (49). Moreover, as noted above, late GBRs are related to higher-order cognitive functions, such as memory and attention, including the updating of memory contents, selection of different behavioral responses, reallocation of attention, or any combination of these (27). Considering these findings, the latent abnormal neural processing of positive information may lead to various cognitive dysfunctions in judgment, memory retrieval, emotion regulation, and attentional orientation, which in turn may underlie cognitive reactivity. Given that the participants with rMDD in this study were fully recovered, it is possible that the late GBRs to positive words reflect the “scars” of depression and serve as persistent markers of the vulnerability to depression recurrence.

While the current study was not designed to determine the cause of the altered late GBRs to positive words in the rMDD group, we can make some speculations based on previous findings of functional changes in the brain. One recent study reported that compared with healthy controls, unmedicated recovered patients with a history of MDD showed attenuated neural responses to pleasant stimuli in the ventral striatum and elevated neural activity in the caudate nucleus in response to aversive stimuli (53). These results indicate that participants with rMDD might have impairments in the neural circuits for reward, and this deficit in the circuits for reward may extend to responses to positive stimuli.

No significant between-group differences in GBRs to negative words were observed in the present study. This conflicts with the findings of Siegle et al. (32), who showed greater sustained gamma activity to negative stimuli in individuals with MDD than in controls. This discrepancy may be explained by differences in the presence of current depressive symptoms. We recruited individuals who had recovered from depression, while Siegle et al. (32) recruited patients in the midst of a current major depressive episode. Some evidence also suggests that individuals with clinical depression show biased attention, memory, and processing for negative stimuli, with specific neural mechanisms that putatively underlie these biases (54). Therefore, rMDD may have led to dysfunctional information processing that differs from that caused by active depression, which may explain these apparently inconsistent results. That said, we focused on two relatively short time windows, that is, early GBRs (50–150 ms) and late GBRs (300–800 ms), whereas Siegle et al. (32) utilized sustained gamma-band activity (0–8000 ms) to evaluate sustained semantic information processing. As our study and that by Siegle et al. (32) examined different neural processes, they cannot be directly compared. Further research is required to more fully investigate the role of late GBRs in people with rMDD vs. MDD.

This study had several limitations. First, we could not determine whether the characteristics of the identified late GBRs were specific to people with rMDD, because we did not directly compare them to those of the late GBRs from different clinical groups, such as individuals with recurrent or single-episode MDD, schizophrenia, or a personality disorder. Future research should aim to identify the neural processes that are specific to rMDD, as this may permit the identification of a neurophysiological biomarker for MDD recurrence. Second, most of the participants with rMDD were young adults (21.72 years old on average), and as a result, they reported a relatively short duration of depressive episodes (7.71 months on average). Considering that the characteristics of cognitive dysfunction in rMDD have been found to vary according to age (55) and the depressive episode duration (56), this may limit the generalizability of our findings to other age ranges and patients with other rMDD severities. Finally, we did not include participants with a history of comorbid disorders and early life stress; however, MDD frequently co-occurs with anxiety (57), and early life stress has been suggested to affect the risk and protective factors of depression (58). Future studies should include more diverse samples to examine whether our findings may be influenced by a previous history of anxiety and early life stress.

## Conclusions

The present study is the first to report altered induced gamma-band activity during the judgment of positive information in people with rMDD. Our findings demonstrate that the greater power of late GBRs to positive words after negative mood elicitation was related to higher cognitive reactivity. This indicates that latent abnormalities in higher-order neural processes, including memory, and attention, might underlie the cognitive reactivity in individuals with rMDD. Overall, these findings suggest that late GBRs are persistent markers of cognitive reactivity, and may therefore be a useful index for evaluating the risk of depression recurrence. Nevertheless, additional research is needed to investigate the specific role of late GBRs in rMDD and compare it to that in other clinical conditions.

## Data Availability Statement

The raw data supporting the conclusions of this manuscript will be made available by the authors, without undue reservation, to any qualified researcher.

## Author Contributions

TY, NS, HK, and HS conceived and designed the experiments. TY and NS performed the experiments. TY, GS, and MT analyzed the data. TY, NS, GS, HK, HS, SM, EM-R, NR, AN, MT, YO, and SY wrote the paper, contributed to and have approved the final manuscript.

### Conflict of Interest Statement

The authors declare that the research was conducted in the absence of any commercial or financial relationships that could be construed as a potential conflict of interest.
